# Signalling through cerebral cavernous malformation protein networks

**DOI:** 10.1098/rsob.200263

**Published:** 2020-11-25

**Authors:** Valerie L. Su, David A. Calderwood

**Affiliations:** 1 Department of Pharmacology, Yale University School of Medicine, PO Box 208066, 333 Cedar Street, New Haven, CT 06520, USA; 2 Department of Cell Biology, Yale University School of Medicine, PO Box 208066, 333 Cedar Street, New Haven, CT 06520, USA

**Keywords:** cerebral cavernous malformation, signalling, protein, subcellular localization

## Abstract

Cerebral cavernous malformations (CCMs) are neurovascular abnormalities characterized by thin, leaky blood vessels resulting in lesions that predispose to haemorrhages, stroke, epilepsy and focal neurological deficits. CCMs arise due to loss-of-function mutations in genes encoding one of three CCM complex proteins, KRIT1, CCM2 or CCM3. These widely expressed, multi-functional adaptor proteins can assemble into a CCM protein complex and (either alone or in complex) modulate signalling pathways that influence cell adhesion, cell contractility, cytoskeletal reorganization and gene expression. Recent advances, including analysis of the structures and interactions of CCM proteins, have allowed substantial progress towards understanding the molecular bases for CCM protein function and how their disruption leads to disease. Here, we review current knowledge of CCM protein signalling with a focus on three pathways which have generated the most interest—the RhoA–ROCK, MEKK3–MEK5–ERK5–KLF2/4 and cell junctional signalling pathways—but also consider ICAP1-β1 integrin and cdc42 signalling. We discuss emerging links between these pathways and the processes that drive disease pathology and highlight important open questions—key among them is the role of subcellular localization in the control of CCM protein activity.

## 1. Introduction

Cerebral cavernous malformations (CCMs) are vascular abnormalities found predominantly in the central nervous system, where they are the second most common type of vascular lesion, comprising 5–15% of all neurovascular malformations [1–3], and having a prevalence of 0.1–0.8% in the general population [3–6]. They are characterized by leaky vessels which exhibit low pressure and low flow, contributing to dark red, blood filled vascular lesions with a mulberry like appearance [6–8]. Within CCMs, endothelial cells lack intervening tight junctions, are surrounded by diminished lamina, collagen and elastic tissue, and have fewer neighbouring pericytes, astrocytes and vascular smooth muscle cells, all of which contribute to thin and leaky vascular walls that are prone to rupture [3,7–9]. Although these lesions can occur anywhere in the body, those in the brain or spinal cord are the most clinically relevant [10] with approximately 40% producing symptoms which include seizures, focal neurological symptoms (e.g. double vision, nausea and mobility problems), haemorrhages or headaches [11–14]. Among these, haemorrhages are the most detrimental symptom and often require surgical resection for treatment [15]. As an improved understanding of the molecular causes of CCM lesion genesis and progression is likely to be instrumental in devising non-surgical strategies to control CCM disease, there have been extensive efforts to identify genes driving the disease, the cellular signalling pathways that are perturbed and the cell structures and interactions that are altered. There are a number of recent reviews on various aspects of CCM [9,16–21]; therefore, here we focus on recent progress on the interactions of CCM proteins and their control of key signalling pathways, as well as potential cross talk between these pathways.

## 2. Identification of genes mutated in CCM

The first major molecular insights into CCMs came from analysis of inherited disease-causing mutations. Between 20% and 50% of CCM cases are familial [22,23] and, compared with sporadic CCMs which typically exhibit a single lesion, familial cases are generally more severe, often presenting with multiple lesions, earlier onset and increased haemorrhage rates [22,24]. CCM1, the first gene linked to CCM disease, was initially mapped to a 4-cM segment of human chromosome 7 [25] and ultimately identified as the gene encoding the protein KRIT1 [26,27]. The identification of kindreds with CCM disease but no *CCM1* mutations [28] led researchers to pursue additional CCM disease-associated genes and the *CCM2* gene was linked to 7p15-p13 and the *CCM3* gene to 3q25.2-27 [29]. These genes were eventually revealed to be the protein-coding genes CCM2 [30] and CCM3/PCDC10 [31]. Loss-of-function mutations in these genes, including nonsense, frameshift and splice site alteration resulting in a premature stop codon, are the most common; however, large deletions and insertions have also been identified [15,32] along with rarer point mutations [33–36]. Inheritance is autosomal dominant, while sporadic cases most likely arise from a germline mutation or a somatic mutation in a single cell [33,37]. In nearly all familial cases, and in about two-thirds of sporadic cases, mutation in at least one CCM gene has been identified, with overall mutation rates ranging between 53–65% for *KRIT1*, 15–19% for *CCM2* and 10–16% for *CCM3* [23,38], confirming the central roles these genes have in CCM disease [39–41].

Lesion genesis is thought to arise from a two-hit mechanism, where loss of both copies of a CCM gene must occur. In most inherited cases, a germline, familial mutation is accompanied by a second somatic, sporadic local hit to remove the remaining wild-type copy leading to homozygosity at the CCM locus [34,42]. Mouse models support this hypothesis as while KRIT1^−/−^ mice exhibit general developmental arrest after E9.5 and die by E11 with severe vascular defects associated with abnormal angiogenenic remodelling, including vascular dilations related to altered arterial fate and elevated endothelial mitosis [43], heterozygous *KRIT1* knockout mice do not normally recapitulate CCM disease phenotypes [42]. However, when crossed into a *p53* knockout background to increase the rate of somatic mutations, heterozygous null KRIT1 mice develop lesions resembling human CCMs [44,45]. Similar findings were reported when heterozygous CCM2 or CCM3 mice are crossed into a *p53* knockout background [45,46]. Likewise, heterozygous *KRIT1^+/−^* mice in a mismatch repair-deficient *Msh2^−/−^* background also display CCM lesions [42]. Notably, at late stages, these lesions exhibit characteristics consistent with human CCMs such as: haemosiderin deposits, immune cell infiltration, increased ROCK activity and increased endothelial cell proliferation [42]. Thus, local loss of both copies of the CCM genes drives pathology. Although *KRIT1*, *CCM2* and *CCM3* are broadly expressed, the cell type most strongly linked to CCM lesions is the endothelial cell; indeed, endothelial-specific deletion of *KRIT1*, *CCM2* or *CCM3* in mice results in lesions in the brain neurovasculature, mimicking human CCMs [43,47,48]. The roles of CCM genes in non-endothelial cells (e.g. neuroglia, pericytes, smooth muscle cells, astrocytes) are much less studied and their contributions to CCM disease are unclear, as while loss of neural CCM3 does produce dilated vasculature and vascular lesions in mice [49], neural or smooth muscle-specific deletion of CCM2 does not recapitulate CCMs [48,50]. For this reason, while acknowledging the importance of the further study of multiple cell types, in this review, we will focus primarily on the potential functions of CCM gene products in endothelial cells and the signalling pathways they use to regulate vascular stability.

## 3. The CCM proteins

The three CCM genes, *KRIT1*, *CCM2* and *CCM3*, encode unrelated adaptor proteins, raising the question of how the loss of any one of these different proteins produces very similar disease phenotypes. The answer likely lies in the ability of these three proteins to assemble into a tri-molecular complex (the CCM complex) and of the complex or the independent proteins to regulate interacting pathways that influence CCM pathogenesis. Here, we provide a brief description of each protein with an emphasis on its domain architecture and binding interactions with proteins likely to influence CCM signalling.

### 3.1. KRIT1

**Figure 1.**
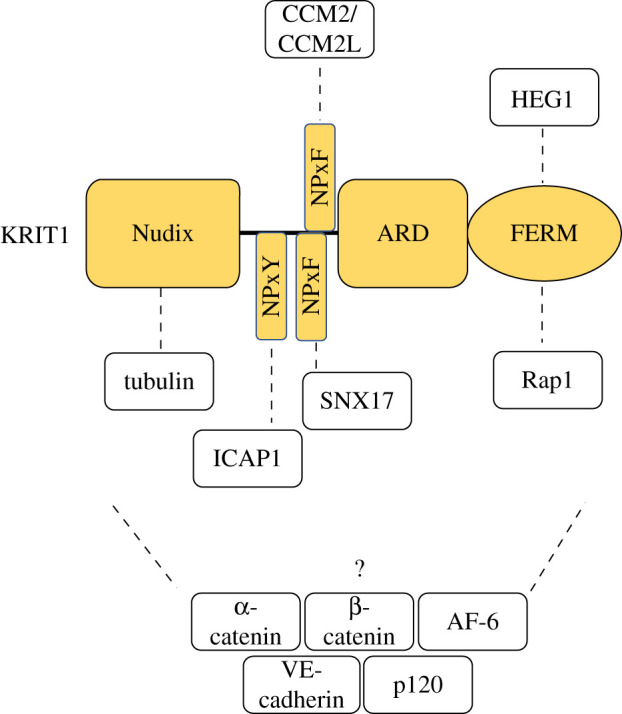
Domain architecture and interaction partners of KRIT1. KRIT1 comprises a Nudix domain, three NPx(Y/F) motifs, an ankyrin repeat domain (ARD) and a FERM (band 4.1, ezrin, radixin, moesin) domain. Tubulin binds KRIT1's Nudix domain, ICAP1 binds KRIT1's 1st NPxY motif, SNX17 binds KRIT1's 2nd NPxY motif, CCM2 and CCM2L bind KRIT1's 3rd or potentially 2nd NPxF motif, and HEG1 and Rap1 bind KRIT1's FERM domain. The binding site of α-catenin, β-catenin, AF-6, VE-cadherin and p120 to KRIT1 or whether the interaction is direct or indirect is currently unclear.

KRIT1 is a 736 amino acid protein and over the past decade crystallography has established that it consists of a N-terminal Nudix domain, three Asn-Pro-*X-*Tyr/Phe (NPx(Y/F)) motifs, a C-terminal ankyrin repeat domain (ARD) and FERM (band 4.1, ezrin, radixin, moesin) domain module [51–56] (figure 1). The domain architecture points to an adaptor protein role for KRIT1 as NPxY/F motifs, ARDs and FERM domains are all recognized as interaction modules. The discovery of an N-terminal Nudix domain was unexpected [53], but the KRIT1 Nudix appears to lack the hydrolase activity normally associated with Nudix domains and might more accurately be described as a pseudo-Nudix. While its function remains unknown, like other pseudo-enzymes [57,58], it seems likely to mediate interactions with other binding partners.

KRIT1 was first identified as a Rap1-binding protein in a yeast two-hybrid screen [59]. Binding occurs via the KRIT1 FERM domain [55] and, as discussed in later sections, this allows Rap1 to localize KRIT1 to junctions where Rap1, KRIT1, and associated proteins exert junction stabilizing activity both by activating junctional tension and inhibiting radial tension [60,61]. The KRIT1 FERM also binds the cytoplasmic tail of the transmembrane protein HEG1 (figure 1) and this, in association with Rap1 binding, contributes to junctional stability and signalling. KRIT1 may also associate with VE-cadherin, α-catenin, β-catenin, AF-6 and p120-catenin at cell–cell junctions influencing junctional stability and β-catenin-mediated transcription [61–63]. However, the nature of these KRIT1 interactions, whether direct or indirect, remain to be resolved.

The KRIT1 NPxY/F motifs support binding to the phosphotyrosine binding domain (PTB) domains of ICAP1 and CCM2 [52,53]. CCM2 binding involves the second or third NPxY/F motifs but ICAP1 binding is restricted to the first NPxY/F motif (figure 1). This interaction stabilizes both KRIT1 and ICAP1 proteins, impairs the ability of ICAP1 to inhibit integrin adhesion receptor activation [53,64,65] and results in ICAP1-mediated nuclear import of KRIT1 [64,66].

In addition to the well-characterized interactions of KRIT1 with ICAP1, CCM2, HEG1 and Rap1, direct interactions with microtubules, possibly via a loop in the KRIT1 Nudix domain, have been postulated [67,68]. The ARD also contains evolutionarily conserved surface patches that suggest potential additional binding sites [51], thus there may be more to be learned about KRIT1 interactions. Furthermore, despite the extensive advances revealing KRIT1 domain architecture, we still lack structures of the intact protein and the potential for conformational regulation of KRIT1 interactions remains [51–53].

### 3.2. CCM2

**Figure 2.**
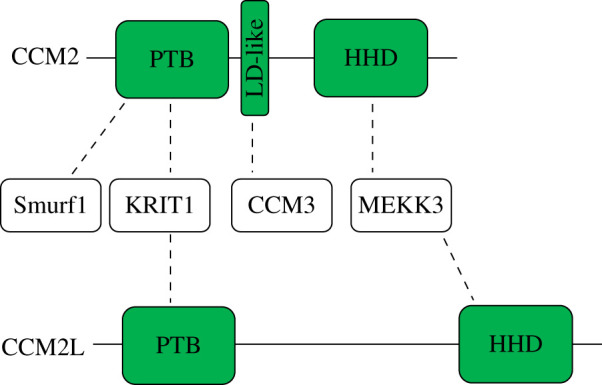
Domain architecture and interaction partners of CCM2 and CCM2L. CCM2 comprises a N-terminal phosphotyrosine binding domain (PTB) domain, a middle LD-like motif and a C-terminal harmonin homology domain (HHD). Smurf1 and KRIT1 binds CCM2's PTB domain, CCM3 binds CCM2's LD-like motif and MEKK3 binds CCM2's HHD. CCM2L comprises a PTB domain and HHD. KRIT1 binds CCM2L's PTB domain and MEKK3 binds CCM2L's HHD.

CCM2 is a 444-amino-acid protein made up of an N-terminal PTB domain, a C-terminal harmonin homology domain (HHD) and a middle LD-like motif linking the two domains [52,69] (figure 2). CCM2 directly interacts with KRIT1 through CCM2's PTB domain and KRIT1's 2nd or 3rd NPx(Y/F) motifs [52] while the CCM2 LD-like motif binds the FAT-H domain of CCM3 [69]. CCM2, therefore, bridges both KRIT1 and CCM3 supporting the assembly of a CCM protein complex. Interactions between the CCM proteins are important for protein stability [62,65,69] and, at least in the case of CCM2–CCM3, interactions are required for normal endothelial network formation [69]. The HHD of CCM2 binds the N-terminus of MEKK3, an upstream kinase in the p38 and ERK5 MAP kinase (MAPK) pathways and this interaction appears necessary to prevent hyperactive MEKK3–MEK5–ERK5 signalling which contributes to CCM pathogenesis [70–72]. Mechanistically, how CCM2 binding influences MEKK3 signalling is currently unknown.

Like KRIT1 knockout mice [43], *CCM2^−/−^* mice are embryonic lethal while *CCM2^+/−^* mice reveal the expected CCM lesion phenotype [48,73]. In 2012, a paralogue of CCM2, CCM2-like (CCM2L) was identified [74]. CCM2L has a similar domain organization to CCM2 (figure 2) and, like CCM2, CCM2L uses its PTB domain to bind KRIT1 and its HHD to bind and inhibit MEKK3 [75], but notably is unable to bind CCM3 [74]. Also like CCM2, CCM2L mediates cardiovascular development as CCM2L deficiency in mice and zebrafish causes perturbed circulation, a ‘big heart’ phenotype, and dilated atrial and inflow tracts [75,76]; however, it is unclear whether CCM2 and CCM2L have redundant, opposing or unrelated roles. For instance, CCM2 and CCM2L compete for binding KRIT1 and MEKK3 [74,75], and while loss of CCM2L in animal models may recapitulate phenotypes resembling those of CCM2 loss, unlike CCM2*^−/−^* mice, *CCM2L^−/−^* null mice grow to maturity in normal numbers and CCM2L appears to stabilize angiogenesis since loss of CCM2L increases lumen formation in endothelial cells [74]. Whether differences relate to competition between CCM2 and CCM2L for binding to KRIT1 or to an inability of CCM2L to recruit CCM3 to the complex is yet to be resolved, but CCM2 and CCM2L may have complementary roles in CCM signalling.

### 3.3. CCM3

**Figure 3.**
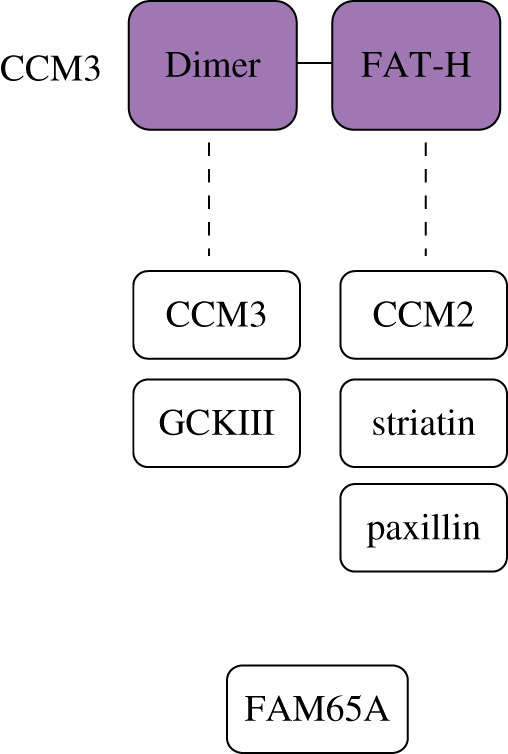
Domain architecture and interaction partners of CCM3. CCM3 comprises a N-terminal dimerization domain and C-terminal focal adhesion targeting-homology (FAT-H) domain. Another CCM3 protein and GCKIII binds CCM3 through its dimerization domain, while CCM2, striatin and paxillin bind CCM3 through its FAT-H domain. Binding of FAM65A and whether this is direct or indirect is currently unclear.

CCM3 (also called PDCD10) is a 212-amino-acid protein made up of a N-terminal dimerization domain and C-terminal focal adhesion targeting-homology (FAT-H) domain [77,78] (figure 3). CCM3 was the first of the CCM proteins to be crystalized and this revealed that the dimerization domain allows homodimerization [77]. The functional relevance of CCM3 homodimers is not clear but the dimerization domain alternatively supports heterodimerization with the germinal centre kinase III (GCKIII) proteins [79], suggesting important roles for CCM3 in GCKIII signalling. The FAT-H domain can bind to many partners containing LD-like motifs, including CCM2, striatin and paxillin but also potentially to phosphotidylinositides [69,80–83]. The CCM2–CCM3 interaction stabilizes both proteins and is important for endothelial network assembly [69] but proteomic experiments suggest that CCM3 mostly resides in the striatin-interacting phosphatase and kinase (STRIPAK) multiprotein complex [82]. There, together with its interacting partners, it plays roles in vascular development, cell cycle control, cell migration and vesicular trafficking [84,85]. For example, CCM3 loss in cells impairs repositioning of both the Golgi complex and centrosome towards the leading edge leading to inhibition of cell migration [85,86], while increased CCM3 expression increases cell migration [86]. Global or endothelial deletion of *CCM3* in mice results in embryonic lethality due to vascular developmental defects and loss of VEGFR2 signalling [87]. Notably, human CCM3 mutations have the most severe clinical consequences [23,46] often with early onset of clinical features and an increased CCM burden [46,88]. These features may suggest that loss of CCM3 leads to CCM pathogenesis through a different mechanism than that of KRIT1 and CCM2. For instance, only CCM3 inhibits exocytosis of angiopoitin-2 (ANG-2) from endothelial cells, and *CCM3* knockout leads to increased ANG-2 secretion resulting in cavernoma development due to decreased endothelial cell adhesion and pericyte coverage [89,90]. Only CCM3 associates with VEGFR2, reducing its endocytosis [91], and CCM3 is required for normal endothelial cell proliferation [69]. However, loss of CCM3, like loss of KRIT1 or CCM2, impacts several core CCM signalling pathways arguing for a common mechanism of pathogenesis that may be exacerbated in the absence of CCM3.

## 4. CCM signalling pathways

Each of the CCM proteins forms distinct macromolecular complexes with other proteins allowing them to impact numerous signalling pathways [9,17,80,84,92–94]. The perturbation of several of these pathways has been strongly linked to CCM disease, but the molecular bases of these connections have not always been fully elucidated. Here, we focus on the well-established MEKK3–MEK5–ERK5–KLF2/4, RhoA–ROCK and junctional signalling pathways while acknowledging other pathways that have also been implicated.

### 4.1. MEKK3–MEK5–ERK5–KLF2/4 signalling

**Figure 4.**
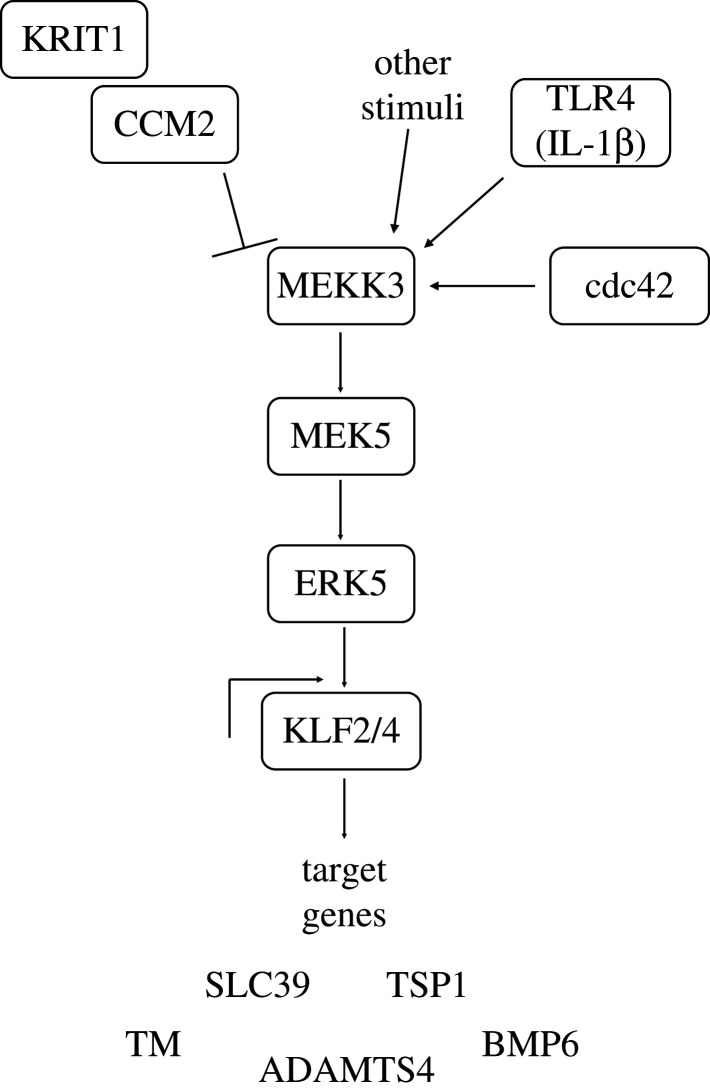
The MEKK3–MEK5–ERK5–KLF2/4 signalling pathway. Loss of KRIT1 or CCM2, activation of cdc42 or TLR4, or other stimuli can result in hyperactivation of the MEKK3–MEK5–ERK5 kinase cascade, leading to upregulation of KLF2 and KLF4 transcription factors and changes in expression of their transcriptional targets such as ADAMTS4, thrombomodulin (TM), thrombospondin 1 (TSP1), bone morphogenic protein 6 (BMP6) and potentially SLC39.

Hyperactivation of the MEKK3–MEK5–ERK5 kinase cascade, leading to upregulation of KLF2 and KLF4 (KLF2/4) transcription factors and changes in expression of their transcriptional targets such as ADAMTS4, thrombomodulin (TM), thrombospondin 1 (TSP1), bone morphogenic protein 6 (BMP6) and potentially SLC39 (figure 4), is now recognized as a key step in CCM disease [16,71,95–99].

Mitogen-activated protein kinase kinase kinase 3 (MEKK3; gene name *MAP3K3*) is a 622-amino-acid protein consisting of a N-terminal helix, a Phox/Bem1p (PB1) domain and a C-terminal kinase domain [70], and is part of both the ERK5 (figure 4) and the p38 MAPK stress-activated protein kinase cascades. MEKK3 is necessary for proper cardiovascular development since heart-specific *Map3K3* knockout mice show embryonic lethality prior to E12.5 [95], and endothelial-specific knockout is neonatal lethal due to multiple intercranial haemorrhages [70]. However, MEKK3 hyperactivation is also detrimental and is observed in cells lacking KRIT1, CCM2 or CCM3 [70,71,75,95]. Importantly, the PB1 domain of MEKK3 directly binds the HHD of CCM2 and a disease-causing human CCM2 mutation abrogates the MEKK3 interaction without affecting CCM complex formation [70,95]. At the molecular level, how CCM2 binding alters MEKK3 activity is unknown, but MEKK3 haplo-insufficiency rescues the loss of cardiac jelly and changes in gene expression conferred by endocardial KRIT1 deletion [95], and also rescues cardiac defects in CCM-deficient embryonic mouse and fish hearts [71]. Furthermore, Ponatinib, a MEKK3 inhibitor, can prevent the formation of new CCM lesions, reduce the growth of already formed lesions and normalize expression of KLF2/4 in neonatal mouse models of CCM [99]. These studies strongly suggest that the CCM2:MEKK3 interaction normally inhibits MEKK3 signalling, preventing downstream upregulation of KLF2/4 and that this is lost in CCM pathogenesis. Consistent with this idea, MEKK3 activation downstream of Toll-like receptor 4 (TLR4) stimulation by lipopolysaccharide derived from the gut microbiome enhances CCM lesion formation and inhibition of TLR4 signalling or altering the microbiome protects KRIT1 endothelial knockout mice from developing CCM lesions [100].

MEKK3 signalling leads to ERK5 activation and its translocation to the nucleus where it drives expression of the transcription factors KLF2 and KLF4. These are known to regulate crucial endothelial responses to pressure and inflammation, and global deletion of either gene in mice results in embryonic lethality or death shortly after birth with vascular defects including gross haemorrhaging [101–103]. The inducible endothelial-specific knockout of *KLF2* and *KLF4* also results in vascular barrier disruption, systemic coagulopathy and rapid death of adult mice [103]. Several high-impact studies have linked overexpression and nuclear translocation of KLF2/4 to MEKK3 activation downstream of KRIT1, CCM2 or CCM3 loss [71,95,104]. In neonatal mouse models of CCM disease, endothelial loss of *MEKK3*, *KLF2* or *KLF4* prevents lesion formation and rescues lethality [71,104]. Furthermore, genetic inactivation of *KLF4* blocks the formation of CCM lesions and abrogates the mortality of mice with endothelial-specific ablation of *KRIT1* by 75% [104]. Therefore, KLF2 and KLF4 are upregulated in CCM lesions and in KRIT1, CCM2 or CCM3 deficient endothelial cells, and their downregulation can reverse CCM disease phenotypes.

Several KLF2/4 target genes have been implicated in driving CCM disease phenotypes; the relative importance of these and whether this varies by cell type is yet to be fully resolved. One gene, *Thbd*, encodes the endothelial anti-blood clotting membrane protein TM [98]. TM levels are increased in human CCM lesions and in the plasma of CCM patients, while, in mice, endothelial-specific deletion of KRIT1 or CCM3 results in KLF2/KLF4-mediated increased levels of vascular TM and endothelial protein C receptor (EPCR) [98]. Importantly, blocking antibodies against TM and EPCR significantly reduce CCM haemorrhage in CCM3 endothelial-specific knockout mice [98]. Consistent with this, genetic inactivation of 1 or 2 copies of the *Thbd* gene decreases brain haemorrhage in these mice [98]. KLF2/KLF4 pathway activation downstream of *KRIT1* gene inactivation also leads to the downregulation of TSP1 (gene name *Thbs1*), a potent endogenous angiogenesis inhibitor [97]. This results in heightened VEGF signalling and weakened tight junctions. Notably, these phenotypes are reversed by *in vitro* reconstitution with full-length TSP1 or with an anti-angiogenic TSP1 fragment [97]. *In vivo*, inactivation of 1 or 2 copies of *Thbs1* aggravates CCM lesion genesis and pathogenesis [97] while administration of the anti-angiogenic TSP1 fragment prevents the development of lesions in *KRIT1* endothelial-specific knockout mice [97]. Thus, normalization of TSP1 levels may improve CCM patient outcomes. The extracellular protease ADAMTS4, which is important in cardiac development [95], and the growth factor BMP6, which may contribute to endothelial-to-mesenchymal transition (EndMT), are also upregulated downstream of MEKK5 signalling in CCM disease [104]. More recently, studies in *Caenorhabditis elegans* and zebrafish revealed KRIT1 and CCM2 regulation of KLF-induced expression of a zinc transporter SLC39, with important non-cell autonomous effects on apoptosis [105], and studies in mice showed KLF2/4-driven induction of microRNA-27a (miR-27a), a negative regulator of VE-cadherin expression [106]. Preventing miR-27a interaction with VE-cadherin mRNA restored endothelial barrier function *in vitro* and normalized vasculature and reduced lesion formation and growth in CCM mouse models [106]. Thus, while some key outputs from the ERK5–KLF2/4 pathway relevant for CCM have been worked out, additional targets of interest are still being identified.

### 4.2. RhoA–ROCK signalling

KRIT1, CCM2 and CCM3 deficient endothelial cells and human CCM lesion samples display activated RhoA and increased activity of downstream effectors ROCK1 and ROCK2, which consequently phosphorylate myosin light chain (MLC) and MLC phosphatase, leading to inhibition of the latter [62,73,107–109]. This increased RhoA–ROCK signalling results in actomyosin contractility and stress fibre accumulation, impairing migration, invasion and 3D tube formation, and destabilizes endothelial adherens junctions, thereby reducing endothelial barrier function and increasing vascular permeability [62,65,73,108,110,111]. Notably, pharmacologic inhibition of ROCK reverses the increased MLC phosphorylation, actin stress fibres and monolayer permeability seen in KRIT1, CCM2 or CCM3 deficient endothelial cells [62,73,108,110], and also rescued the impaired pulmonary and cerebral vascular leak in *KRIT1^+/−^* and *CCM2^+/−^* heterozygous mice [62,73]. Similarly, the ROCK inhibitors, fasudil and atorvastatin, have recently been shown to reduce lesion burden in *CCM3* deficient mice [112]. Thus, loss of KRIT1, CCM2 or CCM3 hyperactivates RhoA–ROCK signalling, and RhoA/ROCK inhibitors may be a viable pharmacologic treatment option for CCM disease.

The exact molecular mechanisms by which loss of KRIT1, CCM2 or CCM3 results in RhoA/ROCK activation is uncertain. The PTB domain of CCM2 has been reported to bind the homologous to the E6-AP C terminus (HECT) domain of the E3 ubiquitin ligase Smurf1, leading to colocalization of Smurf1 and CCM2 at the cell periphery and peripheral degradation of RhoA [113]. Thus, in CCM lesions, loss of CCM2 may reduce Rho degradation leading to hyperactivation of the ROCK pathway, however, despite the appeal of this model, no additional support has been reported in the decade since its initial publication. More recently, the novel Rho effector FAM65A has been shown to associate with CCM3 and the CCM3 binding partners GCK kinases MST3 and MST4 [114]. Loss of CCM3, MST3 and MST4 all result in activated RhoA/ROCK signalling [109], but whether the CCM3:FAM65A interaction influences RhoA or ROCK activity is still unexplored. Several links between KRIT1 loss and RhoA/ROCK hyperactivation have been postulated, including that KRIT1 loss destabilizes CCM2 [65,115] resulting in Smurf1 mislocalization and loss of Rho degradation [113]. As discussed in more detail below, KRIT1 loss also prevents Rap1 stabilization of Rasip1 and its binding partner the RhoA GTPase activation protein, ARHGAP29, at endothelial cell junctions, resulting in increased stress fibre formation and cell contractility [61,116,117]. In addition, KRIT1 co-immunoprecipitates with both ROCK isoforms, ROCK1 and ROCK2, but apparently impacts their functions differently [8,10]. KRIT1 recruits ROCK2 to VE-cadherin junctions but inhibits ROCK1 kinase activity to ensure proper adhesion to the extracellular matrix and stabilize cell–cell junctions [108]. The molecular basis for these isoform-specific effects is currently unclear, but while ROCK2 knockdown phenocopies the heart defects of *kri-1* null zebrafish and the abnormal F-actin and focal adhesion morphologies of KRIT1 and CCM2 null endothelial cells, ROCK1 knockdown restores cortical actin organization and rescues cardiac cushions and ventricular chamber formation in *kri-1* mutant zebrafish [108]. Finally, loss of KRIT1 destabilizes ICAP1 [65], which has also been reported to bind ROCK1 and to localize it to membrane ruffles [118]. How this impacts ROCK1 activity was not addressed. Thus, while Rho/ROCK signalling is clearly enhanced in CCM, the pathway or pathways causing the enhancement have not yet been definitively established.

### 4.3. Junctional signalling

**Figure 5.**
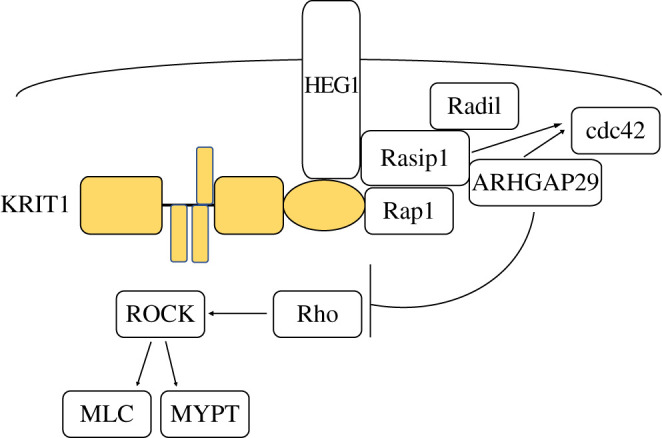
Junctional signalling. KRIT1 forms a complex with Rap1, HEG1 and Rasip1 that is important for their recruitment to and stabilization of endothelial junctions. Rasip1 also binds Radil1 and ARHGAP29 and leads to their recruitment to junctions. This leads to inhibition of Rho activity by ARHGAP29, inhibiting ROCK activation and downstream MLC phosphorylation.

KRIT1 binds the small GTPase Rap1 and directs its localization to cell junctions where, together with other junctional proteins including Rasip1, HEG1, VE-cadherin and β-catenin, they stabilize junctions [56,60,61,63,119,120]. The loss of this targeting is reported to lead to the leaky vasculature phenotype seen in CCM lesions [60,61]. The importance of both isoforms of Rap1 (Rap1a and Rap1b) for vascular stability and haemostasis is well recognized and *Rap1a* and *Rap1b* double knockout mice are embryonic lethal and display haemorrhages at mid-gestation [121–123]. The Rap1/KRIT1 interaction is necessary to stabilize cell–cell junctions since both high expression of Rap1 and disrupting the Rap1/KRIT1 interaction results in the failure of endothelial cells to organize correctly and the formation of enlarged blood vessels [60,61]. It is likely that at junctions KRIT1 and Rap1 are part of a larger complex with the endothelial-specific Rap1 and Ras effector protein Rasip1 [120] and the transmembrane protein HEG1 which binds Rasip1 and KRIT1 only if they are bound to active GTP-Rap1 [56,120,124] (figure 5). HEG1 is highly expressed in the endothelium and *Heg1^−/−^* global knockout mice and zebrafish display defective heart, blood vessel and lymphatic vessel integrity [125,126], while mice lacking Rasip1 have impaired blood vessel tubulogenesis and lumen formation [117].The formation of a Rap1–KRIT1–HEG1–RASIP1 complex (figure 5) is key for recruitment to and stabilization of endothelial junctions, thereby supporting cardiovascular development [120,124].

KRIT1 has also been shown to co-immunoprecipitate with cell-junctional proteins like β-catenin, p120-catenin, α-catenin, AF-6 and vascular endothelium VE-cadherin although the nature of these interactions remains to be resolved [61]. Notably, VE-cadherin is a critical vascular protein involved in CCM signalling [127] and may bind KRIT1 via β-catenin, helping to localize Rap1 to junctions [61,63]. VE-cadherin further controls localization of the Rap1 exchange factors Epac and PDZ-GEF to junctions, supporting local activation of Rap1 and stabilization of endothelial cell barriers [61,128,129].

The Rap1 junctional complex appears to feed into the Rho–ROCK pathway. In addition to binding Rap1 and HEG1, Rasip1 also binds the Rap1 effector Radil and the RhoA RhoGAP ARHGAP29 [116] (figure 5). Therefore, activation of Rap1 not only localizes Rasip1 and HEG1, but also Radil and ARHGAP29, to junctions [61,116]. This leads to inhibition of Rho activity by ARHGAP29, which stimulates GTP hydrolysis converting active GTP-bound Rho into inactive GDP-bound Rho, preventing ROCK activation and downstream MLC phosphorylation [116,117,120,130]. Consistent with this model of cross talk between the Rap1 junctional complex and Rho–ROCK pathway, silencing HEG1 in HUVECs increases MLC phosphorylation and actin stress fibre formation, while re-expressing wild-type HEG1 but not a Rasip1-binding deficient HEG1 mutant reverses this [120]. Knockdown of Rasip1, ARHGAP29 or Radil also increased stress fibre assembly, activated Rho and upregulated phosphorylation of MLC and MYPT (downstream ROCK1/2 substrates), reducing cell spreading and leading to defects in lumen formation and endothelial junctions [117,130]. Notably, these phenotypes can be rescued by RhoA knockdown or treatment with ROCK inhibitors [117,130]. Thus, activation of Rap1 results in localization of KRIT1, HEG1, Rasip1, Radil1 and ARHGAP29 to cell junctions where they stabilize endothelial barriers, by reducing RhoA activity. Consistent with this, knockdown of RhoA results in increased endothelial barrier function and tubulogenesis [130,131], while RhoA overexpression suppresses lumen formation [132,133].

### 4.4. Other CCM-associated signalling pathways and cross talk between pathways

In addition to the three pathways highlighted above, CCM pathogenesis may be associated with perturbed signalling through β1 integrins. Integrins are *αβ* heterodimeric cell surface transmembrane adhesion receptors responsible for cell adhesion to the extracellular matrix [134–136]. The β1 integrin subunit is widely distributed, pairs with multiple different *α* subunits and plays a crucial role in vascular development and angiogenesis [137–140]. Furthermore, the downregulation of β1 integrin rescues CCM mutant malformations in zebrafish [141]. The link from integrins to CCM signalling is likely to be primarily through the β1-integrin- and KRIT1-binding protein ICAP1. Although ICAP1 mutations have not been linked to CCM disease [65], there is abundant evidence that ICAP1 is important for normal vascular development. For example, ICAP1 overexpression in human umbilical vein endothelial cells inhibits endothelial tube formation and sprouting while ICAP1 knockdown enhances these processes [142]. ICAP1-deficient endothelial cells grafted onto the flanks of mice display increased sprouting angiogenesis and denser blood vessel network formation [142] while *ICAP1*^−/−^ blood vessels are more dilated than *ICAP1*^+/+^ blood vessels, and their surrounding basal lamina structure is disrupted [65]. Further, although a subset of *ICAP1*-null mice are viable, they display vascular abnormalities including enhanced dermal bleeding upon dissection and haemorrhagic kidneys, probably due to increased vessel permeability and dilation of the blood vasculature [65].

Yeast two-hybrid screens originally revealed that the PTB domain of ICAP1 bound NPxY motifs in the short cytoplasmic tail of β1 integrins [143,144] and this is now known to inhibit integrin activation, reducing integrin affinity for extracellular matrix [53,64,145]. The PTB domain of ICAP1 also binds the first NP*X*Y motif of KRIT1 [53] (figure 1) and this interaction stabilizes both proteins, preventing their proteasomal degradation [65,115]. The KRIT1 and β1 integrin NPxY motifs compete for binding to ICAP1, thus the ICAP1:KRIT1 interaction can increase integrin activation by displacing the inhibitory ICAP1:integrin interaction [53,64]. Loss of KRIT1 in CCM lesions may, therefore, enhance ICAP1 binding, inhibiting integrin function [53,64]. Alternatively, as loss of KRIT1, and to a lesser extent CCM2, leads to loss of ICAP1, β1 integrin signalling may be activated [65]. Which mechanism is most prevalent is unclear but changes in integrin function may contribute to the altered extracellular matrix remodelling in the lesions of KRIT1 and CCM2-deficient mice [65]. Integrin-ICAP1 signalling is also likely to interface with core CCM pathways as mechanosensing through integrins is required for KLF2 upregulation following loss of CCM proteins, and CCM proteins control endothelial β1 integrin-dependent mechanotransduction in response to shear stress [141,146]. Dysregulated β1 integrin may also link the Rap1/junctional proteins and RhoA–ROCK pathways. Indeed, Rap1 is known to activate integrins in multiple cell types [147–149]. Consistent with this, loss of Rasip1 in endothelial cells strongly reduced β1 integrin activation and adhesion [117]. Conversely, the upregulated RhoA–ROCK signalling in the absence of KRIT1 or CCM2 is proposed to result from β1 integrin hyperactivation as ICAP1-, KRIT1- or CCM2-depleted HUVECs were found to be elongated with transverse bundles of actin stress fibres (both signs of Rho activation), and depleting β1 integrin blocked the formation of these fibres [65]. Additional work will be required to resolve the apparent discrepancy between these two studies, although we note that β1 integrin-mediated adhesion impacts a wide range of cytoskeletal processes making it difficult to directly attribute the increased RhoA–ROCK levels seen in KRIT1 and CCM2-depleted cells to changes in β1 integrin signalling.

Integrins have also been linked to control of signalling through the small GTPase cdc42 [150,151], and cdc42 has recently been linked to CCM [152]. Cdc42 plays crucial roles in the vasculature: its activation is required for lumen formation in 3D collagen matrix assays [117,132,153,154] and its deletion inhibits angiogenesis while inducing aberrant vascular remodelling, defective F-actin organization and disorganized cell–cell junctions [152,155]. Loss of cdc42 also impairs brain endothelial cell sprouting, branching morphogenesis, axial polarity and normal dispersion within brain tissue [152]. A recent report shows that loss of KRIT1 and CCM2 inhibits cdc42, leading to disorganized endothelial junctions and increased vascular permeability [152]. Further, cdc42 is reported to interact with the CCM proteins, and CCM3 promotes cdc42 activity in endothelial cells [152]. In *C. elegans*, CCM3 apparently enhances cdc42 signalling as *ccm-3* null worms have severely reduced cdc42 and active cdc42 protein levels in excretory canals [156]. Further, *cdc-42* ablation by mutation or RNAi causes canal truncations and canal cyst formation similar to those seen in *ccm-3* null *C. elegans* [156]*.* Notably, in mice, post-natal endothelial-specific deletion of *cdc42* elicits malformations reminiscent of CCMs, probably through increased MEKK3–MEK5–ERK5 signalling and consequent increased KLF2/4 [152]. Supporting this mechanism, genetic inactivation of KLF4 attenuates the severity of vascular defects in *cdc42* mutant mice [152].

In addition to acting upstream of MEKK3, cdc42 appears to function downstream of Rap1 and junctional protein signalling. Supporting this model, depletion of the Rap1-binding protein, Rasip1 or its binding partner ARHGAP29, decreased activation of cdc42 and downstream kinases [117] (figure 5). As discussed earlier, the Rap1/junctional protein pathway inhibits RhoA activity, but it appears that these are separate pathways or that cdc42 is downstream of RhoA–ROCK since cdc42-depleted endothelial cells did not significantly change RhoA activation levels [152]. Collectively, junctional proteins appear to decrease RhoA signalling while dysregulating β1 integrin and activating cdc42 signalling thereby protecting against CCM pathogenesis [117].

Although many of the molecular details are lacking, the MEKK3–MEK5–ERK5–KLF2/4 pathway also seems likely to intersect with the RhoA–ROCK pathway and possibly also Rap1/junctional proteins. Inducible endothelial-specific heterozygous loss of *MEKK3*, *KLF2* and *KLF4* in a KRIT1 knockout background reduces CCM lesions, reverses the increase in Rho activation and normalizes MLC phosphorylation, suggesting that changes in RhoA–ROCK activity are downstream of changes in MEKK3 activity [71]. Consistent with this, the Rho/ROCK pathway inhibitors hydroxyfasudil, Tempol and vitamin D3 failed to reverse the increase in KLF2/4 mRNA levels seen in KRIT1-deficient endothelial cells [71]. An increase in KLF2/4 transcription factors leads to reduced TSP1 levels, and TSP1 replacement can prevent CCM disease [97]. Notably, this also restores junctions *in vitro* and *in vivo* suggesting that alterations in cell junctions are also downstream of KLF2/4 targets [97], although roles for alterations in Rap1 signalling have not yet been explored. Levels of the important junctional protein VE-cadherin are also inhibited by miR-27a, whose expression is induced by KLF2/4 downstream of CCM protein loss [106].

In conclusion, many complex protein pathways contribute to CCM pathogenesis, but connections between pathways are gradually being resolved. While in some cases the exact molecular players linking loss of KRIT1, CCM2 or CCM3 to lesion genesis remain unclear, emerging evidence suggests KLF2/4 expression downstream of cdc42, MEKK3 and integrin signalling lies upstream of alterations in junctional proteins and ultimately of Rho activation.

## 5. Subcellular localization of the CCM proteins

The ability of CCM proteins to influence specific signalling pathways depends on their subcellular localization, and cell compartmentalization can provide one method of regulating their functions. Thus, in addition to characterizing CCM protein interactions and signalling pathways, there have been recent efforts to understand CCM protein localization.

KRIT1 has been reported to localize to microtubules [67,68], the cell membrane [56,67,68], cell–cell junctions [60,61,63,120], intracellular vesicles and the nucleus [53,64,66,157]. A polybasic stretch in a loop within the N-terminal Nudix domain of KRIT1 preferentially binds the plus ends of microtubules and Rap1 binding inhibits KRIT1 binding to microtubules, instead allowing KRIT1 recruitment to the membrane and cell junctions [67,68]. CCM2 may influence this distribution as KRIT1 changes from diffusely localized across the nucleus and cytoplasm to more cytoplasmic-localized upon addition of CCM2, which by itself is cytoplasmic-localized [115]. ICAP1 binding is also reported to inhibit KRIT1 binding to microtubules and a ternary ICAP1/KRIT1/Rap1 complex exists *in vitro*, suggesting that this complex may play an important role at the membrane [68]. Notably, both KRIT1 and ICAP1 also have nuclear localizations [53,61,64,66,158], and we find that ICAP1 directs the ICAP1–KRIT1 complex to the nucleus in a manner that requires a nuclear localization sequence (NLS) in ICAP1, but not KRIT1, and which is regulated by ICAP1 serine phosphorylation [64,66]. Nuclear roles for KRIT1 and ICAP1 have yet to be determined, but nuclear KRIT1 localizes to perichromatin fibrils that are sites of active transcription [157] and nuclear ICAP1 potentially binds the c-myc promoter, activating c-myc transcription and driving cell proliferation [158,159]. ICAP1 and CCM2 may, therefore, function to recruit KRIT1 to either the nucleus (when KRIT1 is bound to ICAP1) or cytoplasm (when KRIT1 is bound to CCM2). However, ICAP1, KRIT1 and CCM2 can also exist in a ternary complex, with ICAP1 binding the first KRIT1 NPxY motif and CCM2 binding the 3rd and/or potentially 2nd NPxF motif, and it is possible that this complex shuttles between the nucleus and the cytoplasm to carry out specific subcellular compartmental functions.

KRIT1 has also been reported at intracellular vesicles [68,160]. The second NPxY motif of KRIT1 can bind the FERM domain of sorting nexin 17 (SNX17) [161] and this may partially localize KRIT1 to intracellular vesicles [160]. While it is still unclear what role, if any, KRIT1 plays in intracellular vesicles, we note that CCM3 has been implicated in controlling vesicular traffic [162]. In addition, CCM3 weakly associates with active VEGFR2 at cell membranes in unstimulated conditions and this is strengthened upon VEGF stimulation [87], presumably activating downstream vascular pathways. VEGF triggers endocytosis of the VEGFR2:CCM3 complex from the membrane to intracellular vesicles [87]. Notably, a *CCM3* human disease mutant containing only the N-terminal region is internalized with VEGFR2, even in the absence of VEGF, suggesting that the C-terminal region of CCM3 stabilizes the CCM3:VEGFR2 complex at the membrane [87]. Interestingly, CCM3 is also reported to directly interact with phosphatidylinositol-3,4,5-trisphosphate (PtdIns(3,4,5)P_3_) and to co-localize at the membrane with constitutively active phosphatidylinositol-3 kinase (PI3 K), the enzyme that synthesizes PtdIns(3,4,5) P_3_ [81]. Because VEGF signalling activates PI3 K [163], this suggests a model in which CCM3 interacts with activated VEGFR2 following VEGF treatment, thus activating PI3 K to form PtdIns(3,4,5)P_3_ which can then bind CCM3 to regulate vascular processes [81].

In addition to cell membranes, the major site of CCM3 localization appears to be the Golgi apparatus [85,86] where it seems to carry out functions independent of its binding to CCM2. For instance, unlike CCM2 or KRIT1, CCM3 co-localizes with Golgi protein markers [86] and binds members of the STRIPAK complex including the GCKIII kinases SOK1, MST3, MST4 and striatins, together carrying out CCM3's functions in Golgi repositioning, assembly and cell migration [85,86]. Interestingly, the depletion of CCM3 causes localization of MST4 to the Golgi whereas depletion of striatins prevents MST4's Golgi localization, suggesting CCM3 promotes the cytosolic localization of MST4 [85]. It is not fully clear how the different localizations of CCM3 and its interacting partners affect their roles in Golgi-dependent processes.

The localization of CCM proteins has also been reported to be regulated in response to hyperosmotic shock [72,115]. CCM2 was independently identified as osmosensing scaffold for MEKK3 (OSM) and shown to be important in MEKK3–MEK3 pathway activation of p38 MAPK [72,115]. Hyperosmolarity was also reported to drive CCM2 and MEKK3 localization from the cytosol to actin-rich membrane ruffles [72]. Consistent with this, a separate study also showed that sorbitol-induced hyperosmolarity triggered re-localization of a KRIT1–CCM2 complex from the cytosol to the cell periphery [115] supporting the argument that membrane localization of a KRIT1–CCM2–MEKK3 complex is important for p38 activation. However, as described earlier, there is abundant evidence that CCM2 reduces MEKK3 activation of MEK5 and subsequent ERK5 activation [71,95,100,104,152], and others have reported that knockdown of OSM actually increases p38 phosphorylation of p38, rather than decreasing it [164]. Thus, the roles of CCM protein localization and their consequences on CCM protein signalling are not yet completely understood.

## 6. CCM disease treatment and concluding remarks

As described above, CCM proteins contribute to a series of interconnected signalling networks and are regulated in complex manners, including through control of CCM protein subcellular localization. How these pathways intersect with larger-scale physiological processes, leading to CCM pathogenesis in the absence of CCM proteins, is an important area of ongoing research and continued improvements in understanding CCM signalling pathways should help reveal how molecular changes occurring in the absence of CCM proteins lead to CCM lesion formation and growth. A detailed examination of all potential CCM disease-associated processes is beyond the scope of this review but alterations in oxidative stress, inflammation, the gut microbiome, autophagy, EndMT and angiogenesis have been linked to CCM [16–19,84,94,165]. We note that, consistent with known CCM signalling pathways, changes in endothelial cell junctions, cell contractility and gene expression patterns are central to these processes.

The importance of further advancing understanding of CCM signalling and disease processes is highlighted by the lack of broadly effective pharmacological treatments for CCM disease. Current drug treatment is limited to antiepileptic medications for the subset of CCM patients exhibiting seizures [166], with many of these patients ultimately become resistant to the medication [20]. The most effective method of treatment remains surgery; however, this procedure is risky since remnants may rupture if the lesion is not completely removed [167,168], is limited by lesion accessibility and can be associated with significant rates of morbidity and mortality [169,170]. Insights from analysis of CCM signalling pathways have already led to rationally designed preclinical pharmacological candidates, however, only a few remain in clinical trials [20]. Candidates typically target proteins upregulated or activated as a result of loss of *KRIT1*, *CCM2* and/or *CCM3* in CCM lesions, including ERK [171], EphB4 kinase [172] and TLR4 [100] that are all components of the MEKK3–KLF2/4 axis, as well as fasudil [62,173], atorvastatin [174] and simvastatin [43] which target the RhoA–ROCK axis. Notably, atorvastatin is part of an enrolling phase I/II clinical trial [174]. The anti-VEGF-A antibody bevacizumab [175] and the β-adrenergic blocker and anti-angiogenic agent propranolol are among the few clinical trial candidates [176,177]. While the majority of pharmacological approaches have been rationally designed, recent unbiased small-molecule suppression screens may also hold promise. For instance, a screen using repurposed drug compounds in CCM2-deficient endothelial cells revealed vitamin D_3_ (a physiological compound with autophagy-inducing [178] and antioxidant properties [179] and tempol (a scavenger of superoxide), which were both later shown to reduce lesion burden by approximately 50% in a mouse model of CCM disease [180,181]. Additionally, a screen of 5268 compounds applied to CCM mutant *C. elegans*, zebrafish, mouse or human endothelial cells, identified dozens of new and already identified candidates involved in processes including angiogenesis, innate immunity and the oxidative stress/redox system [182]. Notably, this included indirubin-3-monoxime, a drug originally derived from traditional Chinese medicine, that rescued the CCM phenotype in *kri-1* deficient zebrafish, CCM2 or CCM3-depleted HUVECs and *CCM2* or *CCM3* mutant mice through targeting the MEKK3/KLF2/4 pathway [182]. Thus, unbiased strategies also highlight the importance of CCM signalling pathways identified from the study of CCM proteins.

In summary, there has been rapid recent progress in understanding CCM protein signalling networks, how they impact cellular behaviour, and their intersections with physiological processes. While these advances have yet to be translated into approved therapeutics for CCM disease, both targeted and unbiased approaches, in combination with further investigation of the molecular processes underlying CCM disease, offer hope for pharmacological intervention in this potentially devastating disease.

## Data accessibility

This article has no additional data.
